# Simultaneous silencing of multiple RB and p53 pathway members induces cell cycle reentry in intact human pancreatic islets

**DOI:** 10.1186/1472-6750-14-86

**Published:** 2014-10-11

**Authors:** Stanley Tamaki, Christopher Nye, Euan Slorach, David Scharp, Helen M Blau, Phyllis E Whiteley, Jason H Pomerantz

**Affiliations:** Department of Surgery, Division of Plastic and Reconstructive Surgery, University of California, San Francisco, CA 94143 USA; QB3 East Bay Innovation Center, 2929 7th St, Berkeley, CA 94710 USA; Prodo Laboratories, 32 Mauchly, Irvine, CA 92618 USA; Baxter Laboratory for Stem Cell Biology, Department of Microbiology and Immunology, Institute for Stem Cell Biology and Regenerative Medicine, Stanford University School of Medicine, Stanford, CA 94305 USA; Mohr Davidow Ventures, Menlo Park, CA 94025 USA; Departments of Surgery and Orofacial Sciences, Division of Plastic and Reconstructive Surgery, Craniofacial and Mesenchymal Biology Program, Eli and Edythe Broad Center of Regeneration Medicine, University of California, San Francisco, CA 94143 USA

**Keywords:** Intact human islets, siRNA, Electroporation, RB, p53, Cell cycle reentry

## Abstract

**Background:**

Human pancreatic islet structure poses challenges to investigations that require specific modulation of gene expression. Yet dissociation of islets into individual cells destroys cellular interactions important to islet physiology. Approaches that improve transient targeting of gene expression in intact human islets are needed in order to effectively perturb intracellular pathways to achieve biological effects in the most relevant tissue contexts.

**Results:**

Electroporation of intact human cadaveric islets resulted in robust and specific suppression of gene expression. Two genes were simultaneously suppressed by 80% from baseline levels. When multiple (up to 5) genes were simultaneously targeted, effective suppression of 3 of 5 genes occurred. Enzymatic pretreatment of islets was not required. Simultaneous targeting of RB and p53 pathway members resulted in cell cycle reentry as measured by EDU incorporation in 10% of islet nuclei.

**Conclusions:**

At least three genes can be effectively suppressed simultaneously in cultured intact human pancreatic islets without disruption of islet architecture or overt alterations in function. This enabled the effective modulation of two central growth control pathways resulting in the phenotypic outcome of cell cycle reentry in postmitotic islet cells. Transient exposure to multiple siRNAs is an effective approach to modify islets for study with the potential to aid clinical applications.

## Background

We report the simultaneous suppression of multiple genes in intact adult human pancreatic islets. The study of human islets is hindered by obstacles including difficulty of maintaining islet cells *in vitro* but within their native complex tissue environment, the intact islet, and difficulty modulating gene expression in a majority of islet cells without significantly damaging islet viability, function and architecture. The complexity of islet biology results in part from the physical interactions and communication between the various islet cell types [1–4]. This intercellular network is disrupted when islets are dissociated and cultured, which is a commonly used approach to study islet cells that facilitates transfection or infection with virus (reviewed in [5]). By contrast, modification of islet cell gene expression in the context of intact intercellular relationships should increase the relevance of the information obtained. Thus, the establishment of techniques that expand our ability to study intact islets is merited.

Translational efforts towards clinical applications using intact islets could also benefit from the advances described here. As transplantation of intact islets advances in clinical trials, clinical applications requiring direct modification of gene expression to improve islet survival and engraftment would likely rely on transient modifications that do not result in permanent integration of genetic constructs. In the case of islet transplantation the period of ex vivo culture prior to transplantation affords an opportunity for transient delivery of siRNA to modulate gene expression in efforts to improve transplantation protocols [6, 7]. Moreover, the importance of developing protocols that facilitate mechanistic studies using human islets is underscored by the observations that insights gained from studies of rodent islets often do not translate directly when applied to human tissue [8, 9].

Islet study also has inherent technical challenges shared by other cell types. Although it has been demonstrated that single molecules can significantly influence human beta cell mitogenic responses [10], the ability to commandeer positive and negative proliferation pathways requires modulation of multiple genes at a time. This is in part due to inherent genetic redundancy that often makes single gene modulation insufficient, and also to the need to evaluate upstream and downstream events relating to an initial perturbation. For example, the retinoblastoma (RB) pathway contains three partially redundant pocket proteins, pRB, p107 and p130 in addition to multiple upstream and downstream modulators and effectors [11]. A generally assumed limitation of RNA interference experiments has been the number of targeted genes that can be directly suppressed at one time. In islets, to date single genes have been efficiently suppressed by electroporation [12]. However, whether it is possible in intact islets to efficiently silence multiple genes simultaneously, which may be obligatory to promote robust biological endpoints remains unknown. A number of potentially complicating factors include delivery of adequate amounts of siRNA molecules into individual cells, saturation of the RISC complex, and toxicity to individual cells throughout the islet.

Here we use electroporation to suppress gene expression using siRNA in intact human islets. We show that suppression of multiple transcripts can occur simultaneously using an approach that preserves morphology, viability and glucose responsiveness. Suppression of genes within the RB and p53 pathways illustrates the capacity to use siRNA to dissect functional interactions among multiple pathway members. These findings should prove useful, as they demonstrate a potent and safe approach to transiently modulate gene expression within the native islet environment.

## Results

We sought to determine an optimal method for modulation of gene expression in intact adult human islets that would be transient and would be minimally disruptive in terms of islet architecture, function and viability. In order to be a sufficiently potent approach to achieve biological endpoints, another goal was to successfully modulate more than a single gene at a time. Prior studies by others have shown that adenoviral infection can target the surface cells of the islet, but not the core [13–17], and it has recently been shown that enzymatic partial disruption of islets with accutase followed by electroporation was effective for suppressing expression of a single gene [12].

**Figure 1:**
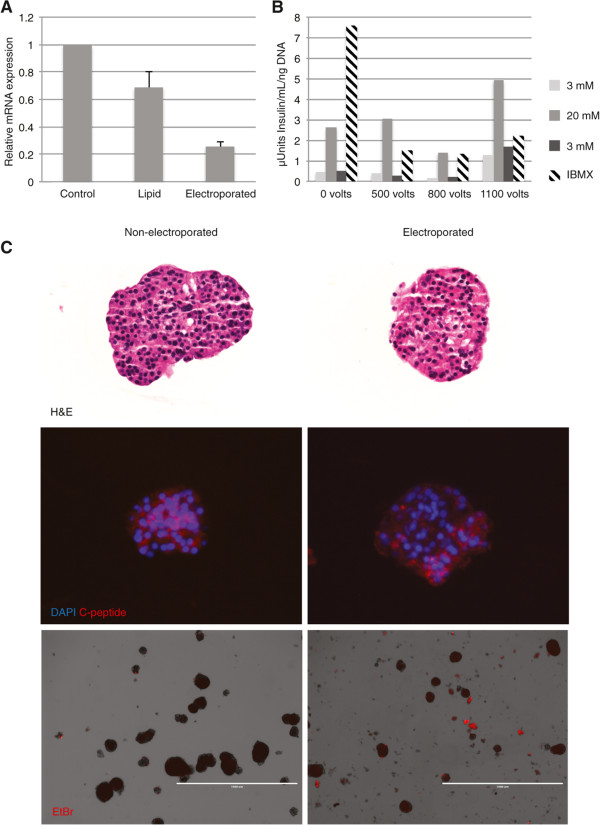
**Electroporation and siRNA delivery in intact human islets. (A)** Graph showing RT-qPCR results of *RB* knockdown following lipid or electroporation transfection. Intact human islets were transfected with non-targeting control siRNA (D-001810 ON-TARGETplus from Thermo Fisher/Dharmacon) or siRNA targeting *RB* (L-003296) using lipid (Dharmafect 1 from Thermo Fisher/Dharmacon) or electroporation (Neon Transfection System from Life Technologies), cultured for two days, and RT-qPCR was performed to assess knockdown. Error bars indicate SEM (n = 3 separate experiments). **(B)** Graph showing representative results of glucose stimulation of insulin secretion assay. Intact islets were electroporated with control, non-targeting siRNA at a range of voltages (x-axis) and a glucose stimulation of insulin secretion assay was performed 72 hours following electroporation. Media was collected after exposure to 3 mM glucose (light gray bars), then after switch to 20 mM glucose (gray bars), followed by return to 3 mM glucose (black bars), and total insulin (hatched bars). **(C)** Images showing morphological comparison between non-electroporated (left) and electroporated (right) islets. Images of islets stained with hematoxylin and eosin (top), immunostained for for c-peptide (red) and nuclei DAPI (blue)(middle), and after exposure to ethidium bromide to test viability 72 hours after electroporation (bottom).

We initially evaluated suppression of a single gene *RB*, using standard lipid based transfection (Figure 1A, and see Table 1 for PCR primers and probes used throughout). Evaluation of gene expression compared to control using quantitative RT-PCR showed modest suppression of total RNA *RB* transcript levels by about 30% relative to controls. This could reflect poor transfection efficiency or inability of the lipid reagent to penetrate the core of the islet. In light of prior reported success with electroporation we explored this approach. We used a capillary tip based system (Neon) to electroporate islets in suspension. Multiple parameters of voltage and pulse times were evaluated first for their effects on islet morphology and viability, and then for gene suppression (data not shown). Ultimately we found that with this system voltages of 1000-1100 volts and a single 40 msec pulse preserved islet morphology and achieved robust suppression of *RB* gene expression by 78% relative to controls (Figure 1A). This robust silencing was attained without digestion of the islet membrane with proteases. Therefore in subsequent experiments we avoided accutase treatment in order to maximally preserve native architecture and islet cell interactions. This level of suppression of *RB* transcript levels indicates both good efficiency of siRNA penetration into islet cells as well as targeting of a majority of cells within the islets.Although a range of voltages and pulse times were effective for suppression, voltages in the 1000-1100 range using a single pulse of 40 msec resulted in robust gene knockdown and spared islets from marked morphological and functional changes. To determine whether electroporation significantly altered islet function we evaluated insulin secretion in response to glucose stimulation. Similar to untreated islets, islets electroporated at various voltages including 1100 volts responded to glucose stimulation by secreting insulin, and the insulin secretion returned to baseline upon exposure to 3 mM glucose (Figure 1B). We did observe lower total insulin levels in the electroporated islets compared to non-electroporated controls. It is possible that this could reflect a decrease in insulin production by islets as a result of electroporation. We found that within this relatively narrow range of electroporation parameters, normal islet morphology was essentially preserved (Figure 1C top panels) and the typical cell types remained, including c-peptide-expressing beta cells (Figure 1C middle panels). Finally, we assessed islet viability after electroporation by ethidium bromide incorporation, and found that although electroporation caused a modest increase in the number of non-viable islets, the majority of islets (75-80%) remained impermeable, indicating good viability (Figure 1C bottom panels). Thus, glucose stimulation of insulin secretion, morphology and cell type composition are preserved after electroporation, supporting the utilization of this approach as a practical method for gene suppression experiments that is minimally disruptive to islet structure and function.

**Table 1:** **TaqMan PCR primers and probes**

Primer/Probe	Sequence
RB F1	5′- GGAAGCAACCCTCCTAAACC - 3′
RB R1	5′ - TTTCTGCTTTTGCATTCGTG - 3′
RB probe	5′ - [6-FAM] CATCTCCCAGGAGAGTCCAA [BHQ1a-6FAM] - 3′
p107 F1	5′ – AGAATGCCTCCTGGACCTTT - 3′
p107 R1	5′ – GGGGTGTCACGAGTGAACTT - 3′
p107 probe	5′ - [6-FAM] ACGCAGAAGAGGAAATTGGA [BHQ1a-6FAM] - 3′
p130 F1	5′ – ATTTGGCATGGAAACCAGAG - 3′
p130 R1	5′ – GTCACCCTTCTGGGAGTCAA - 3′
p130 probe	5′ - [6-FAM] AGAACCTGGAAAGGGCAGAT [BHQ1a-6FAM] - 3′
p21 F1	5′ – AGAGGAGGCGCCATGTCAG - 3′
p21 R1	5′ – CATTAGCGCATCACAGTCGC - 3′
p21 probe	5′ - 6-FAM] CAGAACCCATGCGGCAGCAA [BHQ1a-Q] - 3′
p53 F1	5′ – GTGGAAGGAAATTTGCGTGT - 3′
p53 R1	5′ – CCAGTGTGATGATGGTGAGG - 3′
p53 probe	5′ - [6-FAM] ACATAGTGTGGTGGTGCCCT [BHQ1a-Q] - 3′
ARF F1	5′ – AGGGTTTTCGTGGTTCACAT - 3′
ARF R1	5′ – CTGCCCATCATCATGACC - 3′
ARF probe	5′ - [6-FAM] CGATCCAGGTCCATGATGATG [BHQ1a-6FAM] - 3′
GAPDH F1	5′ – GAGTCAACGGATTTGGTCGT - 3′
GAPDH R1	5′ – TTGATTTTGGAGGGATCTCG - 3′
GAPDH probe	5′ - [6-FAM] CTGAGAACGGGAAGCTTGTC [BHQ1a-6FAM] - 3′

**Figure 2:**
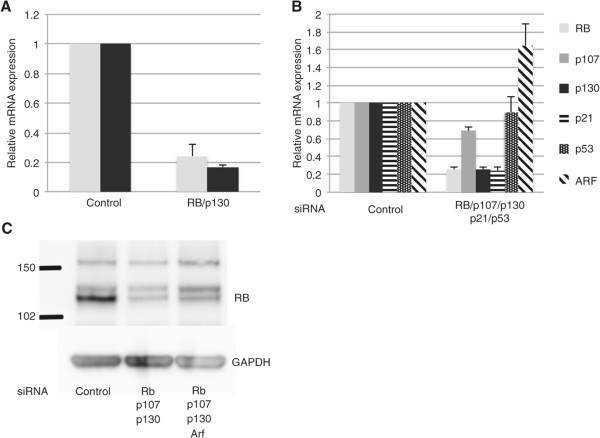
**Simultaneous knockdown of multiple genes in primary human islets following electroporation. (A)** Graph shows RT-qPCR results of islets electroporated with non-targeting control siRNA or siRNA targeting *RB* and *p130* (L-003299). **(B)** Graph shows RT-qPCR results of islets electroporated with non-targeted control siRNA or siRNAs targeting the indicated gene products. The housekeeping gene *GAPDH* was used as the control. Representative experiment shown from 3 separate experiments. Error bars indicate standard deviation. **(C)** Western blot for RB protein in human islet lysates after electroporation of control or targeting siRNA. GAPDH was used as a loading control. RB appears as a characteristic doublet representing the hypo and hyperphosphorylated protein. Representative experiment shown from 3 different experiments.

The potent suppression of a single gene transcript using electroporation prompted us to examine whether this approach would be useful for targeting multiple transcripts simultaneously and thereby enable the perturbation of multiple interacting gene products in the RB and p53 pathways. We found 25 nM to be the optimal concentration of each siRNA for individual gene products. When two distinct gene products were targeted simultaneously, each with a 25 nM concentration of specific siRNA, suppression of mRNA transcript levels was similarly robust as when a single gene was targeted (Figure 2A). Specific siRNAs targeting the pocket protein gene products *RB* and *p130* when combined each at a concentration of 25 nM, and electroporated into intact human islets (1000 volts 40 msec single pulse) resulted in suppression of *RB* and *p130* mRNA levels by approximately 80% of baseline levels when compared to islets electroporated with control siRNA at the same concentration.

Major growth control pathways such as RB and p53 contain multiple upstream and downstream members as well as redundant family members that can compensate for loss of one another [18]. Such complexity significantly complicates experiments designed to study pathway function and presents a challenge for developing practical applications that require modulation of one or more pathways. We targeted various combinations of the RB and p53 pathway members, including *RB*, *p130, p107, p53,* and *p21* each with validated siRNAs (Dharmacon) at 25 nM or 50 nM concentrations, and also measured *p14ARF* transcript levels for changes in response to modulation of other genes. Indeed, we detected a modest induction of *ARF* gene expression after suppression of RB and p53 pathway members, an expected effect based on the known function of *ARF* to detect aberrant RB/p53 pathway signaling. Quantitative RT-PCR showed that at least 3 out of 5 targeted gene products were suppressed to levels considered standard in siRNA experiments. For example *RB*, *p130* and *p21* levels were suppressed by approximately 80% compared to control levels in samples treated for *RB, p130, p107, p53* and *p21* (Figure 2B)*.* In multiple experiments (Figure 2B and data not shown) suppression of 4-5 genes resulted in variability in terms of reduction of each targeted mRNA. Likely contributing factors include saturation of the intracellular RNA interference machinery or induction of gene expression by activation of compensating pathway members in response to suppression of others (known to occur among the pocket proteins as well as among the p53 pathway members). We did not observe any significant increased toxicity when using higher total siRNA concentrations (up to 300 nM) required for these experiments. To confirm that electroporation of siRNA and gene suppression measured by mRNA was an indicator of decreased protein levels, Western blotting for RB protein was performed after treatment with control siRNAs and after combinations of targeting siRNAs. We found expected suppression of RB protein as suggested by the mRNA data, confirming the validity of the PCR data (Figure 2C).

**Figure 3:**
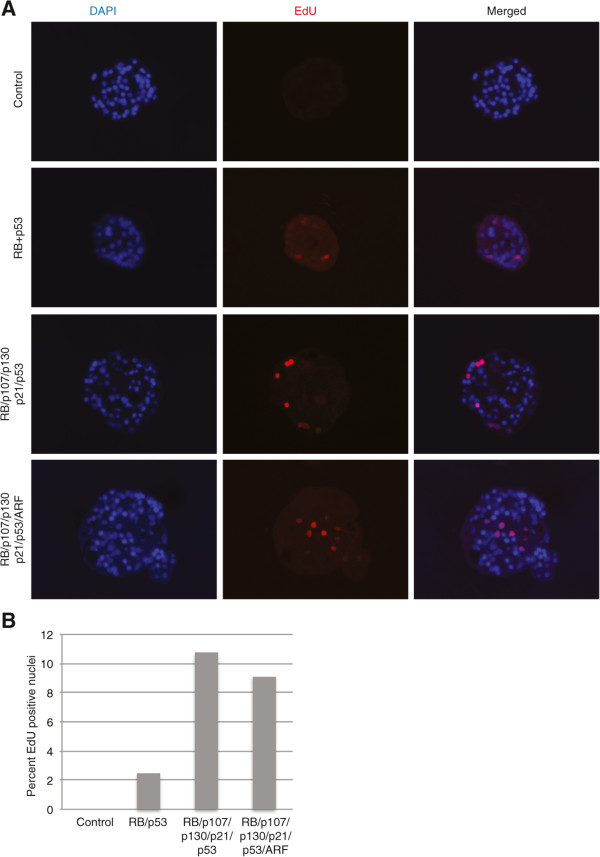
**Cell cycle reentry in primary human islets following knockdown of RB and p53 pathway members.** Primary human islets were electroporated with siRNAs against non-targeting control, or combinations of RB, p107 (L-003298), p130 (L-003299), p21 (L-003471), p53 (L-003329) and ARF/CDKN2A (L-011007). Islets were cultured in the presence of 5-ethynyl-2′-deoxyuridine (EdU) for 7 days following electroporation then fixed and stained with DAPI and for EdU. **(A)** Images showing EdU incorporation in Islets with nuclei stained with DAPI (blue, left), EdU (red, middle) and a composite image (right). **(B)** Graph showing percentage of EdU incorporating cells in islets following transfection. Total cells counted in the representative experiment presented are: siControl (245); Rb + p53 (535); Rbfamily + p53 + p21 (456); Rbfamily + p53 + p21 + Arf (834). The experiment was repeated 3 separate times.

The ability to suppress multiple genes simultaneously using electroporated siRNA indicated that this approach might be useful for functional modulation of the RB and p53 pathways. We tested this by attempting to target multiple interacting genes simultaneously in the RB and p53 pathways to achieve the phenotypic endpoint of cell cycle reentry in mature post mitotic islet cells. This would indicate reversal of relatively stringent maintenance of the islet postmitotic state. After exposure to various siRNA combinations (Figure 3), islets were incubated in 5-ethynyl-2′-deoxyuridine (EdU) for 5 days. Whereas islets treated with control siRNA did not incorporate EDU and those treated for suppression of only *RB* and *p53* siRNA rarely contained cells that incorporated EDU, those that had targeted multiple pathway members often contained clusters of EDU positive nuclei (Figure 3A). Up to 10% of nuclei incorporated EDU when 5 or 6 genes were targeted (Figure 3B). Together, the experiments targeting multiple members of the RB and p53 pathways clearly demonstrate that by simultaneously targeting several genes, the postmitotic state of human islet cells can be reversed to induce cell cycle reentry as evidenced by DNA synthesis and EDU incorporation. This biological effect was not achieved by suppression of single genes in these pathways, a demonstration of the potential utility of this siRNA combination approach.

## Discussion

One challenge facing the development of therapeutics to treat disorders of pancreatic islets is the difficulty of studying intact human islets. Although many important insights have and will continue to come from studies of rodent islets, the significant differences between islets from rodents and humans and the failure of many rodent findings to be conserved in human islet biology underscores the need for studies using human tissue. For example, adult rodent islets can readily undergo islet expansion in different circumstances including increased beta cell stress from Type 2 Diabetes [19]. But adult human islets are more refractory, and efforts to demonstrate adult beta cell expansion have met with great difficulty [20]. Moreover, rodents are genetically tractable and germline targeting of genes of interest is routine, but the limited lifespan of human islets in culture makes such studies using primary human tissue not possible at present. Therefore there is an unmet need for approaches to modify gene expression in intact human islets for translational application. The present study furthers that effort by demonstrating culture and transfection parameters that permit suppression of multiple genes at once, resulting in a phenotypic outcome not achievable with single gene manipulations.

This study builds on previously established approaches to transfect primary cells including islets using electroporation. In contrast to previous work [12], we used intact human islets transfected without accutase digestion and by modifying the electroporation parameters of voltage and frequency, pervasive suppression of targeted genes could be achieved without enzymatic disruption of the islet architecture. This advance is potentially beneficial because of the comparatively lesser manipulation of islets. Survival of electroporated islets and the maintenance of function after electroporation may have been aided in our study by the use of developed culture conditions and media designed to optimize islet survival and resistance to stress [21].

This study extends previous work [12] by showing that electroporation is a superior means of delivering siRNA into islets than liposome-based transfection. We compared the two approaches directly and electroporation caused a significantly greater reduction in targeted mRNA compared to lipofection. We show here that after optimization of siRNA concentrations and electroporation parameters two simultaneous knockdowns were possible to levels comparable to standards for easily transfected immortalized cell lines. Remarkably, we found that significant robust suppression of at least three genes simultaneously is possible using this method. Despite the preservation of islet structure and glucose responsiveness achieved with the present approach, it is likely that this system could be further optimized, for example by decreasing voltage in combination with use of newer siRNA modifications that endow greater transfection efficiency and potency [22, 23]. It is possible that subtle alterations in islet viability or function not detected in the assays used in this study would be better preserved at lower voltages.

Multiple gene suppression in the absence of damage to islet structure or glucose responsiveness allowed us to effectively target two central growth control pathways with inherent redundancies in each. Simultaneous compromise of the RB and p53 pathways led to entry of postmitotic islet cells into the cell cycle. This phenotypic outcome was a result of specific inhibition of the targeted genes and required suppression of multiple pathway members. These findings demonstrate the potential of transient suppression of multiple genes to have significant functional impact in situations where single gene targeting is inadequate. Future experiments will address global changes in islet differentiated and metabolic state after RB/p53 suppression by electroporation. Electroporation of human islets should prove a useful approach to aid the study of other important pathways containing multiple members.

## Conclusions

The application of electroporation in optimized culture conditions is an effective approach to deliver siRNA molecules targeting multiple genes into a majority of cells within intact human islets. Benefits are that it is transient, avoids genetic integration and is well tolerated by islets. The capacity to significantly impact molecular pathways by targeting multiple genes within them should prove to be a valuable tool for both basic study and for practical applications.

## Methods

### Primary Islets cells and culture conditions

Primary human cadaveric islets were obtained from Prodo Labs. Typically islets were shipped overnight on ice 2-4 days after isolation. Upon receipt islets were diluted 50% in prewarmed 37°C PIM(S) media supplemented with PIM(ABS) and PIM(G) (complete) (all purchased from Prodo Laboratories) and centrifuged for 2 min 1200 rpm. Islets were washed and resupended at a concentration of 300-400 islet equivalents (IEQ) per ml in complete PIM(S) media in non-tissue culture treated flasks. Islets were incubated at 37°C in 5% CO_2_ with 50% media change every other day. For the samples used in our experiments the purity of the islets ranged between 85 and 90% in all samples. The average islet size was: 50-100 microns (74%); 100-200 microns (20%); 200-300 microns (5%); 300 microns and higher (1%).

### siRNAs

All siRNAs were ON-TARGETplus SMARTpools purchased from Dharmacon, Thermo Scientific. The following siRNAs were used:

siControl (D-001810)

RB (L-003296)

p107 (L-003298)

p130 (L-003299)

p53 (L-003329)

p21 (L-003471)

ARF/CDKN2A (L-011007)

### Lipid Transfection of islets

Islet transfection with cationic lipid was conducted by diluting 1 μl of Dharmafect 1 (T-2001 Life Techonogies) in 50 μl of PIM(S) followed by addition of siRNAs in 50 μl of PIM(S), vortexing and then allowing the mixture to sit at room temperature for 20 min. The lipid-siRNA mix was added to 400 IEQ in 400 μl complete media in 24-well low adhesion plates and incubated at 37°C in 5% CO_2_. The next day 1.5 ml of complete media was added to each well. The plates were then returned to the incubator to allow the islets to settle to the bottom and 1.25 ml of media was removed with a pipet. This process was repeated 3 times to remove the cationic lipid-siRNA following which the islets were returned to the incubator.

### Electroporation of islets

Electroporation of islets was conducted using the Neon Transfection System (Life Technologies). Islets were centrifuged for 2 min 1200 rpm and then transferred to a 1.5 ml Eppendorf tube and washed with PBS and re-centrifuged. Islets were resuspended at a density of 400 IEQ per 10 μl in R buffer (Life Technologies) and typically electroporated once at approximately 1000-1100 Volts for 40 msec. For knockdown and EdU incorporation experiments the following concentrations of siRNA were used 25 nM RB, 25 nM p130, 25 nM p21, 50 nM ARF/CDKN2A, 50 nM p53, 50 nM p107 and siControl ranged from 25 nM-300 nM. Each batch of electroporated Islets was cultured individually in a single well of a 24-well low adhesion plates (per manufacturer recommendations) in 0.5 ml complete PIM(S) media. Viability was evaluated using ethidium bromide (Molecular Probes) diluted to 1:1000.

### Evaluation of gene expression

Typically, cellular RNA was isolated from 400 IEQ 72 hours after electroporation using an RNeasy Mini Kit (Qiagen) and used to generate cDNA with SuperScript VILO Master Mix (Invitrogen). 2 μl cDNA was used as template for TaqMan PCR in conjunction with TaqMan Fast Advanced Master Mix (Invitrogen). TaqMan PCR was performed using a StepOnePlus™ Real Time PCR system (Life Technologies) with the following cycling parameters;


All PCR data between samples were normalized to GAPDH expression levels and the level of gene knockdown was expressed relative to siCONTROL sample.

### Western blots

Islet samples for protein analysis were pelleted by centrifugation lysed in RIPA buffer and sonicated. After protein concentration was determined, 30 micrograms of protein were loaded per lane. For detection of Retinoblastoma protein blots were probed with 0.1 μg/mL of Human RB1 (MAB6495, R&D Systems) followed by HRP-conjugated Anti-Mouse IgG Secondary Antibody (R &D Systems). GAPDH was similarly done with 0.05 μg/mL of Mouse Anti-Human GAPDH Monoclonal Antibody (MAB5718, R&D Systems).

### EdU or Insulin staining

Islet assay for DNA synthesis was initiated by switching the culture media 48 hr after electroporation into PIM(R) media supplemented with PIM(ABS) and PIM(G) and 10% FBS (all purchased from Prodo Laboratories) containing 5 μM EdU (Life Technologies). Half the media was replaced each day containing 5 μM EdU. 1000 IEQ equivalents for each sample were harvested 7 days after electroporation by centrifuging for 2 min at 1200 rpm followed by aspiration of the media. The islet pellet was resuspended in the residual media and 25 μl of histogel (60°C) was added, mixed with the islets, and quickly placed on ice. The plug was fixed in 4% paraformaldhye-PBS overnight and then dehydrated in alcohol prior to paraffin embedding (Histotech Lab). Paraffin blocks were sectioned at 5 microns onto glass slides. Sections were deparaffinzed using orange oil and passaged through an alcohol series. EdU incorporation was assayed by staining for EdU using a Click-iT EdU Alexa Fluor 568 Imaging Kit (Invitrogen) according to manufacturer’s instructions. In the case of insulin staining deparaffinzed sections were blocked with 5% goat serum in 0.1% Triton PBS for 30 min and anti-pro-insulin C-peptide (Millipore, CBL94) 1:500 dilution and then incubated overnight. Sections were washed and stained with secondary antibody, goat-anti-mouse Alexa 556 (1:500) was used. For both EdU and antibody staining, cover-slips were mounted on sections with Vectashield Mounting Medium containing Dapi (Vector Lab). Digital photographs were taken using a 20X objective on an EVOS FL Cell Imaging System (Life Technologies).

### Glucose Stimulation Insulin Secretion assay (GSIS)

Approximately 100 IEQ were placed into inserts. A glucose stimulation insulin secretion assay was conducted on islets 48 hr after electroporation. Approximately 100 IEQ were placed into millicell (Millipore Inc.) cell culture inserts in a 24 well plate. The minicell inserts were transferred into new wells containing 3 mM Glucose Media (50% Hams F10 (110 mg/dl glucose) 50% DMEM (no glucose), 7 mM Nabicarbonate, 0.75 mM CaCl_2_ 2H_2_O and 10 μg/ml of Ciprofloxan. The inserts with islets were transferred to wells containing 3 mM Glucose Media for 1-2 min to wash out the original media. To remove insulin from the cultures, the islets were washed three separate times with 3 mM Glucose Media and then incubated overnight in the same media at 37 degrees with 5%CO2. The following morning islets were again washed by two 1 hr incubations in 3 mM Glucose Media before initiation of media collection to assay secretion of insulin into the media. For the first collection of the assay, the inserts plus islets were placed into 1 ml fresh 3 mM Glucose Media and incubated for one hour, and then the media was collected. Further samples were collected after 1 hr incubations in 20 mM Glucose Media. Finally the inserts plus islets were washed 2 times with 3 mM Glucose media and then incubated for a final 1 hr in 3 mM Glucose media and collected. The insert plus islets were then transfer to 10 mM Tris-1 mM EDTA pH 8.0 and islets collected from inserts and centrifuged at 180 g for 5 min. The islets were lysed by resuspension in 10 mM Tris-1 mM EDTA pH 8.0 plus 1% Triton-X containing protease inhibitors and vortexed. All samples collected were stored at -80°C until analysis.

ELISA to detect insulin was conducted using Millipore Human Insulin ELISA kit. The two PIM(S) samples from each matrix condition were pooled and assayed as a single sample. The protocol was followed as suggested by the manufacturer with the following modifications. Samples from 3 mM Glucose media were diluted 1:2, 12 mM Glucose media were diluted 1:5, 20 mM Glucose media diluted 1:10 and lysed islets diluted 1:20 prior to testing in ELISA assay. Plates were read at 450 nm absorbance on an Epoch Biotek plate reader.

DNA from lysed islets was quantitated using Invitrogen Quant-iT PicoGreen dsDNA assay kit. Samples were measured on a PerSeptive Biosytems Cyto-fluor and plotted against a standard curve of lambda DNA.
